# TRAJECTORIES OF DEPRESSIVE SYMPTOMS BEFORE THE ONSET OF DEMENTIA: EXAMINING RACIAL/ETHNIC DISPARITIES

**DOI:** 10.1093/geroni/igad104.1669

**Published:** 2023-12-21

**Authors:** Ji Hyun Lee, Amanda Cook Maher, Jordan Palms, Stipica Mudrazija, William Chopik, Laura Zahodne

**Affiliations:** Montana State University, Bozeman, Montana, United States; University of Michigan, Ann Arbor, Michigan, United States; University of Michigan, Ann Arbor, Michigan, United States; Urban Institute, Washington, District of Columbia, United States; Michigan State University, East Lansing, Michigan, United States; University of Michigan, Ann Arbor, Michigan, United States

## Abstract

Biological and cognitive changes can often be detected up to 16 years prior to a formal diagnosis of Alzheimer’s disease (AD). Psychological symptoms of AD (e.g., depression) may also start to develop during this prodromal phase. Depressive symptoms may be an early marker of neurodegeneration and/or a reaction to early cognitive decline. Higher levels of depressive symptoms could create additional burdens for individuals with preclinical AD and their families. Unfortunately, little is known about the trajectory of depression during the preclinical phase of AD. Using the Langa-Weir method for algorithmic classification, a sample of incident dementia cases (n=2168) was identified from longitudinal data from the Health and Retirement Study (2000-2018). Depressive symptoms were measured with eight dichotomous items from the Center for Epidemiologic Studies Depression (CES-D). Latent growth curve models estimated the trajectory of depressive symptoms over up to 10 waves prior to dementia classification, and disparities across racial/ethnic groups were examined. Results show that depressive symptoms increased slightly over time throughout the preclinical phase of AD, following a quadratic trajectory. Older Hispanic adults reported consistently higher levels of depressive symptoms across timepoints, followed by older non-Hispanic Black and older non-Hispanic White adults. These findings highlight the importance of examining the psychological changes occurring during the preclinical phase of dementia for understanding the disease progression of AD and racial/ethnic disparities in psychological burden. The implications of using algorithmic dementia classifications in studying cognitive race/ethnic disparities will also be discussed.

